# Biomarkers of acute kidney injury in patients with nephrotic syndrome

**DOI:** 10.1590/2175-8239-JBN-2020-0021

**Published:** 2020-09-11

**Authors:** Maria Brandão Tavares, Caroline Vilas Boas de Melo, Paula Neves Fernandes, Maria da Conceição Chagas de Almeida, Marcia Fernanda dos Santos Melo Carneiro, Rilma Ferreira de Souza Santos, Marilia Bahiense-Oliveira, Reinaldo Martinelli, Washington LC dos-Santos

**Affiliations:** 1 Hospital do Subúrbio, Coordenação de Ensino e Pesquisa, Salvador, BA, Brazil.; 2 Fundação Oswaldo Cruz, Instituto Gonçalo Moniz, Salvador, BA, Brazil; 3 Escola Bahiana de Medicina e Saúde Pública, Medicina, Salvador, BA Brazil.; 4 UNIME, Lauro de Freitas, BA, Brazil.; 5 Hospital Geral Roberto Santos, Nefrologia, Salvador, BA, Brazil.; 6 Nefrovita - Dialysis, Lauro de Freitas, BA, Brazil.; 7 Hospital Português, Salvador, BA, Brazil.

**Keywords:** Kidney Tubular Necrosis, Acute, Glomerulonephritis, Kidney Injury Molecule 1, Lipocalin-2, Acute Kidney Injury, Necrose Tubular Aguda, Glomerulonefrite, Molécula de lesão renal 1, Lipocalina-2, Lesão Renal Aguda

## Abstract

**Introduction:**

Emergence of acute kidney injury (AKI) in patients with nephrotic syndrome (NS) requires prompt diagnosis and differentiation between acute tubular necrosis (ATN) and proliferative glomerulonephritis. We studied the potential use of commercial urinary biomarkers' tests in the diagnosis of AKI in patients with NS.

**Methods:**

A cross sectional estimate of urinary concentrations of KIM-1 and NGAL was performed in 40 patients with NS: 9 with proliferative glomerulopathy, being 4 with AKI and 31 without proliferative glomerulopathy, being 15 with AKI. AKI was defined using the KDIGO criteria.

**Results:**

The mean age was 35 ± 16 years. The main diagnoses were focal and segmental glomerulosclerosis (10, 25%), membranous glomerulopathy (10, 25%), minimal change disease (7, 18%), lupus nephritis (6, 15%), and proliferative glomerulonephritis (3, 8%). Patients with ATN had higher levels of urinary KIM-1 (P = 0.0157) and NGAL (P = 0.023) than patients without ATN. The urinary concentrations of KIM-1 (P= 0.009) and NGAL (P= 0.002) were higher in patients with AKI than in patients without AKI. Urinary NGAL and KIM-1 levels were significantly higher in patients with ATN without proliferative glomerulonephritis than in patients with proliferative glomerulonephritis (P = 0.003 and P=0.024, respectively).

**Conclusions:**

Neutrophil gelatinase associated lipocalin (NGAL) and kidney injury molecule 1 (KIM-1) estimates correlated with histological signs of ATN and were able to discriminate patients with AKI even in conditions of NS. Furthermore, urinary levels of NGAL and KIM-1 may be useful in the differential diagnosis of acute tubular necrosis and exudative glomerulonephritis in patients with nephrotic syndrome.

## RESUMO

**Introdução:**

O surgimento de lesão renal aguda (LRA) em pacientes com síndrome nefrótica (SN) requer diagnóstico imediato e diferenciação entre necrose tubular aguda (NTA) e glomerulonefrite proliferativa. Avaliamos o uso potencial de testes de biomarcadores urinários comerciais no diagnóstico de LRA em pacientes com SN.

**Métodos:**

Uma estimativa transversal das concentrações urinárias de KIM-1 e NGAL foi realizada em 40 pacientes com SN: 9 com glomerulopatia proliferativa, sendo 4 com LRA e 31 sem glomerulopatia proliferativa, sendo 15 com LRA. A LRA foi definida usando os critérios da KDIGO.

**Resultados:**

A média de idade foi de 35 ± 16 anos. Os principais diagnósticos foram glomeruloesclerose segmentar e focal (10, 25%), glomerulopatia membranosa (10, 25%), doença por lesão mínima (7, 18%), nefrite lúpica (6, 15%) e glomerulonefrite proliferativa (3, 8 %). Os pacientes com NTA apresentaram níveis mais elevados de KIM-1 urinário (P = 0,0157) e NGAL (P = 0,023) do que pacientes sem NTA. As concentrações urinárias de KIM-1 (P = 0,009) e NGAL (P = 0,002) foram maiores em pacientes com LRA do que em pacientes sem LRA. Os níveis urinários de NGAL e KIM-1 foram significativamente maiores em pacientes com NTA sem glomerulonefrite proliferativa do que em pacientes com glomerulonefrite proliferativa (P = 0,003 e P = 0,024, respectivamente).

**Conclusões:**

As estimativas de lipocalina associada a gelatinase de neutrófilos (NGAL) e molécula de lesão renal 1 (KIM-1) se correlacionaram com sinais histológicos de NTA, e foram capazes de discriminar pacientes com LRA mesmo em condições de SN. Além disso, os níveis urinários de NGAL e KIM-1 podem ser úteis no diagnóstico diferencial de necrose tubular aguda e glomerulonefrite exsudativa em pacientes com síndrome nefrótica.

## INTRODUCTION

Acute kidney injury (AKI) is a common complication in patients with nephrotic syndrome, and it is reported in 20% of adults with minimal change disease^1^. Chen and colleagues (2011) associated acute tubular necrosis (ATN) with AKI observed in the course of idiopathic nephrotic syndrome^2^. In a previous study, we showed that ATN was common in patients with glomerular disease and that the intensity of ATN was correlated with the presence of renal failure^3^. The histological findings of ATN, such as the loss of the brush border on the proximal tubular epithelial cells and single cell necrosis with tubular simplification, are typically patchy, with a focal distribution 4. For this reason ATN lesions can be misinterpreted or overlooked on routine biopsy diagnosis, contributing, at least in part, to the overemphasized idea that a dissociation exists between histological tubular injury and renal failure^4^^,^ 5.

Emergence of AKI in the course of nephrotic syndrome requires a prompt differential diagnosis between ATN and proliferative glomerular lesions leading to rapidly progressive glomerulonephritis. ATN usually requires supportive measures, while proliferative lesions can require immunosuppression 6. Although clinical and conventional laboratory clues can be suggestive in many cases, sometimes such distinctions rely on renal biopsy, which is the gold standard for diagnosis albeit an invasive procedure and not available in many centers. Furthermore, it was shown that nephrotic syndrome required more time to resolve in patients with severe AKI, as defined according to the Risk, Injury, Failure Loss and End-stage kidney disease (RIFLE) classification (Chen et al, 2011)^2^. Such delay in nephrotic syndrome resolution may depend on the underlying cause of AKI. Hence, a requirement exists for new tools allowing for the early diagnosis of AKI and differential diagnosis between ATN and proliferative glomerular lesions as causes of AKI.

Currently, the diagnostic criteria for AKI are defined by elevation in serum creatinine levels and a decrease in urinary output, although serum creatinine is a later marker of kidney dysfunction and does not reflect kidney damage in some instances 7. Several new biomarkers have emerged, increasing the perspective on early diagnosis and the prognostic prediction of AKI. Evidence exists that these biomarkers might also indicate the mechanism of AKI. For instance, the levels of neutrophil gelatinase-associated lipocalin (NGAL), a protein of the lipocalin superfamily expressed in the kidneys and released in urine under ischemic and toxic conditions, have shown good performance in AKI prediction in experimental animal models and in humans^8^^,^ 9. Another promising biomarker is kidney injury molecule-1 (KIM-1), a type-1 membrane protein that is not expressed in normal kidneys but is highly expressed in proximal tubules after ischemic and toxic injury^10^^-^ 12. KIM-1 has an ectodomain that is released into the tubular lumen and can be measured in the urine. Although the performance of these biomarkers has been extensively investigated in intensive care and cardiac surgery patients, their use in the differential diagnosis of AKI in patients with nephrotic syndrome still requires study.

In this work, we studied the use of tests based on the urinary concentrations of KIM-1 and NGAL for the diagnosis of AKI in patients with nephrotic syndrome and examined the association of urinary concentration of these biomarkers with histological lesions consistent with ATN or proliferative glomerular lesions.

## METHODS

**Patients:** This was a cross sectional study in which we reviewed the biopsies and clinical data of patients with nephrotic syndrome subjected to renal biopsies during relapse or initial presentation in the referral hospitals for renal disease in Salvador, Bahia, Brazil, between January 2012 and December 2013. The patients were divided in three groups according to presence of ATN histologically defined and proliferative glomerulonephritis, as follows: control group (CON), patients with neither proliferative glomerulonephritis nor ATN; acute tubular necrosis group (ATN), patients with ATN and without proliferative glomerulonephritis; and proliferative glomerulonephritis group (PGN), patients with proliferative glomerulonephritis or the presence of crescent lesion in renal biopsy, regardless of the presence of ATN. We studied the potential use of urinary estimates of KIM-1 and NGAL in the diagnosis of AKI in patients with nephrotic syndrome and correlated histological lesions of ATN with urinary concentrations of KIM-1 and NGAL.

All of the biopsies were examined at the Instituto Gonçalo Moniz - Fiocruz, Salvador, Brazil. Cases were excluded if the histological slides were not available, if interstitial fibrosis was estimated to encompass 30% or more of the renal cortex area, or if there were fewer than 7 glomeruli in the sample.

**Clinical data:** The following data were obtained from biopsy request forms and from the patients' medical records: age, sex, diagnosis of nephrotic syndrome, serum creatinine, albumin, cholesterol, and triglyceride concentrations, 24-hour urine protein excretion rate, and diagnosis of systemic arterial hypertension. Patients were diagnosed with nephrotic syndrome when the urine protein excretion rate was greater than 3.5 g/24 h associated with edema, hypoalbuminemia and dyslipidemia. AKI was defined as a serum creatinine increase of 0.3 mg/dL or 1.5x the baseline value, according to the KDIGO definition of AKI. Baseline creatinine value was defined as the lowest measurement during the previous year. When previous measurements were not available, the lowest creatinine value estimated during hospitalization was used.

**Histological analysis:** All of the renal specimens were obtained by percutaneous biopsies. They were fixed in Bouin's solution, embedded in paraffin, cut in 2-µm slices, and stained with hematoxylin and eosin. A pathologist (WLCS) reviewed all of the slides. The severity of ATN was estimated as the percentage of the renal cortex that contained tubules with evidence of recent necrosis on examination by conventional optical microscopy. The following tubular changes were considered evidence of recent ATN: tubular dilatation with thinning of the tubular epithelium, accompanied by cellular casts and interstitial edema and the presence of morphological markers of epithelial regeneration, such as nuclear hyperchromatism, mitosis and binucleation. As shown previously, even mild ATN is associated with higher risk of renal failure. Hence, we used the extent of tubular necrosis for separating patients into two groups: with ATN, 10% or more of renal cortex presenting necrotic tubules and without ATN, less than 10% of the renal cortex presenting necrotic tubules. The intensity of tubular interstitial fibrosis was also estimated as a percentage of the renal cortex with increased extracellular matrix by visual assessment.

**KIM-1 and NGAL measurements in the urine:** Morning urine samples were collected prior to renal biopsy. They were centrifuged at 2,000 x *g* at 4°C, and the supernatant was stored in 2-mL aliquots at -80°C. Urinary concentrations of KIM-1 and NGAL were measured by enzyme linked immunosorbent assay (R&D Systems and Bioporto, respectively), according to the manufacturers' instructions. All the samples were run in duplicate.

**Statistical analysis:** Data are reported as absolute numbers or percentages and are summarized as means, standard deviations, or medians, using lower and upper percentiles when appropriate. The performance of the biomarkers estimate for the diagnosis of AKI was assessed by analyzing a receiver operating characteristic (ROC) curve. Comparisons between groups were performed using the t-test or the Mann-Whitney (nonparametric) test when applicable. Differences involving proportions were analyzed using the chi-square test. The results were considered statistically significant if *P* < 0.05. Data were analyzed using Prism software, version 5.01 (GraphPad, San Diego, CA, USA), and Stata IC software (StataCorp LP, College Station, TX, USA), version 11.

**Ethics approval:** The study was conducted in accordance with the resolution No. 196/96 (http://conselho.saude.gov.br/Web_comissoes/conep/aquivos/resolucoes/23_out_versao_final_196_ENCEP2012.pdf) and the resolution No. 039/2011 (http://conselho.saude.gov.br/web_comissoes/conep/carta_circular/Uso_de_dados_de_prontuarios_para_fins_de_Pesquisa.pdf) of the National Health Council, and the procedures were approved by the Ethics Committee for Research Involving Human Subjects of Instituto Gonçalo Moniz, Fiocruz, Protocol No. 188/09 and No. 380/12.

In accordance with these resolutions, written informed consent was obtained from all patients involved in the study. Patient records were only reviewed after approval by the Ethics Committee and authorization by the Hospital Clinical Board as required by the resolution No. 039/2011. All the informed consents are maintained in the records of the Laboratório de Patologia Estrutural e Molecular - LAPEM, Fiocruz Bahia.

## RESULTS

**Figure 1:**
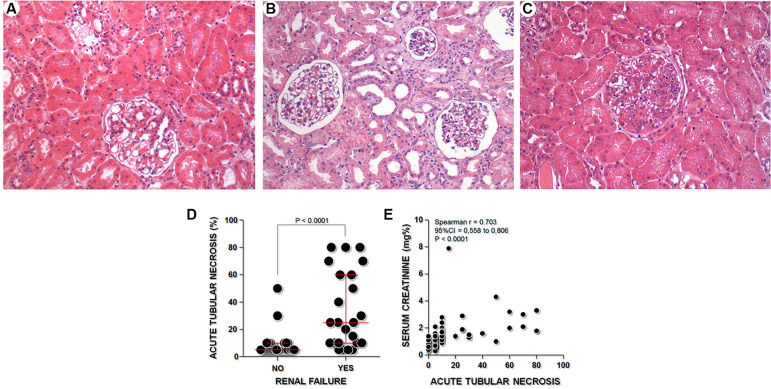
Histological patterns present in the kidney biopsies of the patients of the study: (A) Minimal Change Disease without Acute Tubular Necrosis (ATN). (B) Minimal Change Disease with ATN. (C) Acute Post-infectious Glomerulonephritis without ATN. (H&E x200). (D) Association of renal failure with acute tubular necrosis in patients with nephrotic syndrome (Mann-Whitney test, P <0.0001). (E) Correlation between acute tubular necrosis and serum creatinine concentrations (Spearman, r = 0.703 95% CI = 0.558 to 0.806, P < 0.0001).

Sixty-five patients with nephrotic syndrome subjected to renal biopsies between January 2012 and December 2013 were randomly selected, and clinical data and urine samples were collected prior to renal biopsy. Twenty-five patients were excluded: 20 (30%) had more than 30% cortical fibrosis estimated, and 5 (8%) had fewer than 7 glomeruli in the biopsy. Forty patients were included and the clinical and laboratory data of the three groups are presented in Table 1. The mean age was 35 ± 16 years and 22 (55%) subjects were female. Arterial hypertension was diagnosed in the majority of patients (27; 68%). The main histological diagnoses were focal and segmental glomerulosclerosis (10; 25%) and membranous glomerulopathy (10; 25%) followed by minimal change disease (7; 18%), lupus nephritis (6, 15%), and proliferative glomerulonephritis (3; 8%). The sample comprised patients with nephrotic syndrome with and without proliferative glomerular lesions (Figures 1 A-C). ATN (≥10% of the represented renal cortical area) was present in 19 (48%) of the patients, five of whom also had proliferative glomerulonephritis.

**Table 1:** Comparative characteristics of patients with nephrotic syndrome with and without acute tubular necrosis and with exudative glomerular disease.

Parameter	ATN	CON	PGN	p value
N	14/40 (35%)	17/40 (43%)	9/40 (23)	
Sex:				
Male	6/14 (43%)	9/17 (53%)	3/9 (33%)	0.718
Female	8/14 (57%)	8/17 (47%)	6/9 (67)	
Age:				
Mean ± SD	39 ± 18	37 ± 16	26 ± 11	0.066
Range	15 –62	14 –59	17-53	
Median [interquartile range]	43 [21–52]	35 [21–53]	25 [21-25]	
Creatinine (mg/dL)	1.5 ± 0.8	1.1 ± 0.4	1.2 ± 0.7	0.148
Urea (mg/dL)	74 ± 44	61 ± 46	40 ± 18	0.185
Albumin (g/dL)	1.67 ± 0.28	1.88 ± 0.71	2.21 ± 0.54	0.092
Cholesterol (mg/dL)	376.8 ± 137.5^a^	368.1 ± 88.02^b^	224.4 ± 71.6^a.b^	0.009^a.b^
Urine 24-hour protein (mg) Median [p25-p75]	8399 [3391–15088]	4988 [3276–9040]	2897 [2400-8817]	0.268
ASH	10/14 (71%)	12/17 (71%)	5/9 (56%)	0.753
ACEi/ARB	12/14 (86%)	12/16 (75%)	4/9 (44%)	0.128
Antihypertensive drugs	6/14 (43%)	8/16 (50%)	5/9 (56%)	0.847
Diagnosis:				
MCD	2/14 (14%)	5/17 (29%)		0.371
FSGS	5/14 (36%)	5/17 (29%)		0.371
MGN	3/14 (21%)	7/17 (42%)		0.371
Others	4/14 (29%)*			
LN			6/9 (67%)	
PGN			3/9 (33%)	

Note: CON = control group, ATN = acute tubular necrosis group, PGN= proliferative glomerulonephritis group; ASH, arterial systemic hypertension; ACEi/ARB, angiotensin converting enzyme inhibitor/angiotensin receptor blocker; MCD, minimal change disease; FSGS, focal segmental glomerulosclerosis; MGN, membranous glomerulopathy; LN, lupus nephritis; PGN, proliferative acute glomerulonephritis.

* Others: Amyloidosis (n=1), membranoproliferative glomerulonephritis (n=1), descriptive diagnosis (n=2).

Baseline serum creatinine value was available in 31 patients, of whom 19 (59%) presented with AKI according to KDIGO criteria. In nine patients, the baseline creatinine value was not available. Seven of these nine patients had isolated serum creatinine concentrations in the normal range. When estimated by the isolated serum creatinine concentration on the day of biopsy, 14 (35%) patients presented with renal failure. ATN was associated to renal failure in patients with nephrotic syndrome (P < 0.0001, Figure 1 D) and positively correlated with the concentration of serum creatinine (r = 0.703 95% CI = 0.558 to 0.806, P < 0.0001) (Figure 1 E).

**Urine biomarkers and their associations with AKI in patients with nephrotic syndrome:** The urinary concentrations of KIM-1 and NGAL on renal biopsy had a good performance for indicating AKI based on the KDIGO criteria, with an area under the ROC curve of 0.8000 (95% CI = 0.6328-0.9672) and 0.8647 (95% CI= 0.7068-1.0000) respectively. The urinary concentrations of KIM-1 and NGAL were higher in patients with AKI (KIM-1 = 4254 [815-9534] pg/mL and NGAL = 102.4 [33.50-1195ENT#093; ng/mL) than in patients without AKI (KIM-1 = 1006 [202-2539] pg/mL), P= 0.009; and NGAL = 23.1 [6.6-182.1] ng/mL, P=0.002). Among the patients with proliferative glomerulonephritis, the KDIGO criteria for AKI were applied in only 6 patients, and 4 of them had AKI. Hence, further comparison between this group and the group of patients with AKI without proliferative glomerulonephritis was not performed (Table 2).

**Table 2:** Urinary biomarkers for acute kidney injury (AKI) in patients with nephrotic syndrome: associations with acute tubular necrosis (ATN), proliferative glomerulonephritis (PGN), and AKI defined by KDIGO criteria.

PARAMETER			
	**ATN**	**CON**	**PGN**
NGAL (ng/mL)			
Mean ± SD	381.5 ± 445.7^a^	79.7 ± 149.6	81.9 ± 93.5^a^
Range	22.8 – 1195	6.35 – 587.2	13.1 - 287.9
Median [interquartile range]	146.2 [84.6 – 765]	41 [8.1 – 80.4]	48.9 [21.8 - 126.3]
KIM-1 (pg/mL)			
Mean ± SD	4443 ± 2789^b^	1967 ± 1543	1951 ± 2121^b^
Range	630 – 9534	470 – 5378	202 - 6889
Median [interquartile range]	4884 [1568 – 6224]	1508 [877 – 2539]	1218 [820 - 2294]
	**w/AKI**	**wo/AKI**	
NGAL (ng/mL)			
Mean ± SD	301.8 ± 412.5^c^	43.6 ± 52.7^c^	
Range	33.5 – 1195	6.6 – 182.1	
Median [interquartile range]	102.4 [54.5 – 365.2]	23.1 [11.7 – 61.5]	
KIM-1 (pg/mL)			
Mean ± SD	3993 ± 2778^d^	1226 ± 763^d^	
Range	815 – 9534	202 – 2539	
Median [interquartile range]	4254 [1420 – 5831]	1006 [590 – 2002]	

Note: CON = control group, ATN = acute tubular necrosis group, PGN= proliferative glomerulonephritis group;

^a^ P = 0.0034;

^b^ P = 0.024;

^c^ P 0.002;

^d^ P = 0.009.

Urine biomarkers and their associations with ATN in patients with nephrotic syndrome: Positive correlations were observed of urinary concentrations of KIM-1 (r = 0.4884 95% CI 0.1856 to 0.7065, P = 0.0008) and of NGAL (0.5299 95% CI 0.2290 to 0.7383, P < 0.001) with the percentage of renal cortex with histological lesions of ATN. Patients with ATN ≥10% had higher concentrations of urinary KIM-1 (4299 [630-9534] pg/mL) and NGAL (126.1 [22.8-1195] ng/mL]), compared to patients without ATN (KIM-1=1508 [202-5308] pg/mL, P = 0.0157; and NGAL=41 [6.4-587.2], P = 0.023). Urinary concentrations of NGAL and KIM-1 were significantly higher in patients with ATN without proliferative glomerulonephritis than in those with proliferative glomerulonephritis (NGAL: 146.2 [22.8-1195] vs 48.9 [13.1-287.9], P = 0.00324; KIM-1: 4884 [1568-6224] vs 1218 [820-2294], P=0.024).

## DISCUSSION

In the present study, we found that AKI diagnosed by the KDIGO criteria was frequent in patients with nephrotic syndrome. Appel and colleagues (2007) reported AKI in 24 of 95 adult patients with minimal change disease^1^. AKI was already present in the initial presentation of minimal change disease or during a relapse^1^. Similarly, using the RIFLE criteria, Chen and colleagues (2011) found a 34% incidence of AKI in patients with idiopathic nephrotic syndrome^2^. The frequency of AKI found in our study was higher than that reported by Chen and colleagues (2011). However, because the diagnosis of renal failure might have contributed to the renal biopsy recommendation, the high proportion of cases (59%) reported here might have overestimated the actual prevalence of renal failure in the general population of patients with nephrotic syndrome^2^^,^ 1.

Studies associating the histological patterns of ATN, renal failure, and urinary biomarkers for AKI have been scarce in humans. Most of the knowledge in this field comes from histological studies in experimental animal models, transplanted kidneys, and intensive care patients^13^^,^ 14. Some authors have correlated the KIM-1 and NGAL expression in human kidney biopsies with interstitial fibrosis, inflammation, and the presence of macrophage and myofibroblasts. Van Timmeren and colleagues (2007) showed that urinary levels of KIM-1 were correlated with the tissue expression of the molecule and with the presence of interstitial and glomerular macrophages^15^. However, histological lesion of ATN was not assessed. Because we excluded substantial tubule-interstitial fibrosis from the analysis, the association of histological lesion of ATN with urinary concentrations of KIM-1 and NGAL in humans could be clearly demonstrated. Urine concentrations of KIM-1 and NGAL correlated well with both AKI and ATN in patients with nephrotic syndrome. The high urinary concentrations of AKI biomarkers, even in patients with only 10% of the renal cortex with histological ATN, as shown in the present study, suggested that mild histological lesions may have an impact on the glomerular filtration rate and might reflect more extensive lesions.

Most studies have assessed the performance of urinary NGAL and KIM-1 for predicting the diagnosis and prognosis of AKI in animal models of ischemic and toxic AKI, critical illness, and heart surgery patients^16^. However, few studies have assessed the performance of these tests under conditions of high protein concentrations in the urine. Ogrizovic and colleagues (2013) reported an inverse correlation between the tissue expression of KIM-1 and the estimated glomerular filtration rate years after renal biopsy 17. Different from our study, patients with renal allograft rejection or with diabetic and hypertensive nephropathy were included in Ogrizovic and colleagues' (2013) study, and AKI diagnosis was not assessed. Moreover, nephrotic syndrome was not present in all the patients in their study^17^. Bennett and colleagues (2012) suggested that urinary NGAL levels could distinguish children with steroid-resistant nephrotic syndrome because these children had significantly higher levels of urinary NGAL than controls and steroid-sensitive children^18^. In this study, we showed that estimated urinary concentrations of KIM-1 and NGAL have potential use in the diagnosis of AKI even under the conditions of nephrotic syndrome. No additional step of albumin precipitation was required to perform the tests using commercial kits.

Urinary concentrations of NGAL and KIM-1were higher in patients with ATN associated with non-proliferative glomerulopathy than in patients with proliferative glomerulonephritis. Previous studies have demonstrated induction of NGAL in epithelial cells in inflammatory conditions, and in kidney, there is early expression of NGAL in proximal tubule epithelial cells in ischemic reperfusion injury. In patients with lupus nephritis, urinary NGAL excretion was predictive of the renal disease with better accuracy than Anti-dsDNA, and urinary concentrations of NGAL seem to correlate with disease activity and are elevated in renal flares^19^. In experimental model of antibody-mediated nephritis, the lack of NGAL was associated with attenuated histopathological lesion and kidney dysfunction. However, Eller and colleagues (2013) found that NGAL provides protective effect in murine nephrotoxic nephritis, inducing apoptosis of inflammatory cells; hence, the role of NGAL in pathogenesis of kidney diseases is not well elucidated^20^. The absence of KIM-1 expression in normal kidney, and the expression of this protein in epithelial proximal tubule cells in ischemic or toxic kidney injury suggests that KIM-1 is a specific marker of acute tubular injury. Van Timmeren et al (2007) demonstrated that urinary concentrations of KIM-1 are significantly higher in patients with ATN compared to other kidney diseases^15^. KIM-1 expression was associated with interstitial fibrosis in patients with glomerular diseases, however, in our study, patients with substantial fibrosis in renal biopsy were excluded and there was positive correlation between histological intensity of ATN and urinary KIM-1 concentrations. Therefore, higher urinary concentrations of NGAL and KIM-1 in patients with ATN compared with patients with proliferative glomerulonephritis in this study may be attributed to more severe tubular lesion in ATN group, and not to an under-regulated expression of NGAL and KIM-1 in patients with proliferative glomerulonephritis. Further, prospective studies with larger number of patients might indicate potential cut-off points for urinary biomarker concentrations with potential use in the differential diagnosis between ATN alone and proliferative glomerulonephritis as the cause of AKI in patients with nephrotic syndrome.

Finally, although AKI has been considered an uncommon complication of nephrotic syndrome, recent studies have demonstrated that AKI is, in fact, common in the course of idiopathic nephrotic syndrome in adults with minor glomerular abnormalities. Proteinuria has been associated with impaired antioxidative response, tubular epithelial cell injury, and apoptosis in animal models of tubular necrosis^21^^,^ 22. Although the proximal tubular epithelial cells regenerate and restore kidney function after an AKI episode, a recent epidemiological study showed that AKI was associated with an increased risk of the progression of kidney disease to advanced stages 23. Persistent KIM-1 expression has also been implicated in the progression of kidney disease 24. In an ischemia reperfusion injury model, the NGAL and KIM-1 genes were still upregulated in the kidneys 28 days after ischemic injury, and they were associated with the presence of interstitial fibrosis, suggesting that these markers might indicate the transition from AKI to chronic kidney disease^25^. Phenotypical changes in tubular epithelium and the anomalous transit of molecules to the renal interstitial compartment associated with ATN might contribute to disease progression. The impact of ATN on the prognosis of glomerular disease is not established, and understanding the mechanisms of ATN in patients with non-proliferative nephrotic syndrome could help to define strategies for preventing the progression of kidney lesions toward chronicity. Future studies will be crucial in determining whether the high expression of biomarkers of AKI and consequently the presence of ATN in patients with nephrotic syndrome are associated with worse prognosis and with a higher risk of progression of glomerular disease appearing in patients with nephrotic syndrome.

## CONCLUSIONS

1. ATN is a common cause of renal failure in patients with nephrotic syndrome;
2. Neutrophil gelatinase-associated lipocalin (NGAL) and kidney injury molecule 1 (KIM-1) estimates were correlated with histological signs of ATN;
3. The commercially available kits for NGAL and KIM-1 measurement were able to distinguish patients with AKI from among patients with nephrotic syndrome without requiring further processing steps;
4. Future studies evaluating urinary concentrations of NGAL and KIM-1 could be used to differentiate ATN alone from proliferative glomerulonephritis;
5. The use of these markers does not rule out the requirement of kidney biopsy for the diagnosis of kidney disease in adult patients with nephrotic syndrome.
